# Hydrocarbonoclastic Biofilm-Based Microbial Fuel Cells: Exploiting Biofilms at Water-Oil Interface for Renewable Energy and Wastewater Remediation

**DOI:** 10.3390/bios14100484

**Published:** 2024-10-08

**Authors:** Nicola Lovecchio, Roberto Giuseppetti, Lucia Bertuccini, Sandra Columba-Cabezas, Valentina Di Meo, Mario Figliomeni, Francesca Iosi, Giulia Petrucci, Michele Sonnessa, Fabio Magurano, Emilio D’Ugo

**Affiliations:** 1 Department of Information Engineering, Electronics and Telecommunications, Sapienza University of Rome, Via Eudossiana 18, 00184 Rome, Italy; petrucci.1585491@studenti.uniroma1.it; 2 Department of Infectious Diseases, Italian National Institute of Health, Viale Regina Elena 299, 00161 Rome, Italy; roberto.giuseppetti@iss.it (R.G.); fabio.magurano@iss.it (F.M.); 3 Core Facilities, Italian National Institute of Health, Viale Regina Elena 299, 00161 Rome, Italy; lucia.bertuccini@iss.it (L.B.); francesca.iosi@iss.it (F.I.); 4 Department of Neuroscience, Italian National Institute of Health, Viale Regina Elena 299, 00161 Rome, Italy; sandra.columbacabezas@iss.it; 5 Institute of Applied Sciences and Intelligent Systems, National Research Council of Italy, Via Campi Flegrei 34, 80078 Pozzuoli, Italy; valentina.dimeo@na.isasi.cnr.it; 6 Department of Environment and Health, Italian National Institute of Health, Viale Regina Elena 299, 00161 Rome, Italy; mario.figliomeni@iss.it; 7 Bio-Fab Research Srl, Via Mario Beltrami 5, 00135 Rome, Italy; michelesonnessa@biofabresearch.it

**Keywords:** MFC, water–oil interface, sustainable energy generation, wastewater remediation, oil spill bioremediation, bioelectrochemical systems

## Abstract

Microbial fuel cells (MFCs) represent a promising technology for sustainable energy generation, which leverages the metabolic activities of microorganisms to convert organic substrates into electrical energy. In oil spill scenarios, hydrocarbonoclastic biofilms naturally form at the water–oil interface, creating a distinct environment for microbial activity. In this work, we engineered a novel MFC that harnesses these biofilms by strategically positioning the positive electrode at this critical junction, integrating the biofilm’s natural properties into the MFC design. These biofilms, composed of specialized hydrocarbon-degrading bacteria, are vital in supporting electron transfer, significantly enhancing the system’s power generation. Next-generation sequencing and scanning electron microscopy were used to characterize the microbial community, revealing a significant enrichment of hydrocarbonoclastic *Gammaproteobacteria* within the biofilm. Notably, key genera such as *Paenalcaligenes*, *Providencia*, and *Pseudomonas* were identified as dominant members, each contributing to the degradation of complex hydrocarbons and supporting the electrogenic activity of the MFCs. An electrochemical analysis demonstrated that the MFC achieved a stable power output of 51.5 μW under static conditions, with an internal resistance of about 1.05 kΩ. The system showed remarkable long-term stability, which maintained consistent performance over a 5-day testing period, with an average daily energy storage of approximately 216 mJ. Additionally, the MFC effectively recovered after deep discharge cycles, sustaining power output for up to 7.5 h before requiring a recovery period. Overall, the study indicates that MFCs based on hydrocarbonoclastic biofilms provide a dual-functionality system, combining renewable energy generation with environmental remediation, particularly in wastewater treatment. Despite lower power output compared to other hydrocarbon-degrading MFCs, the results highlight the potential of this technology for autonomous sensor networks and other low-power applications, which required sustainable energy sources. Moreover, the hydrocarbonoclastic biofilm-based MFC presented here offer significant potential as a biosensor for real-time monitoring of hydrocarbons and other contaminants in water. The biofilm’s electrogenic properties enable the detection of organic compound degradation, positioning this system as ideal for environmental biosensing applications.

## 1. Introduction

Harnessing the metabolic activities of microorganisms to convert organic substrates into electrical energy, microbial fuel cells (MFCs) represent a promising technology for sustainable energy generation [1,2]. These bio-electrochemical systems offer a clean and cost-effective power source, and their potential applications span across wastewater treatment [3], desalination [4], and energy production [5]. The principle underlying MFCs involves microorganisms oxidizing organic compounds at the anode, producing electricity, and simultaneously contributing to the breakdown of organic waste [6,7,8,9,10,11].

Recent advancements in MFC technology have improved their efficiency and expanded their potential applications, particularly in wastewater treatment and low-power micro-grids suitable for powering applications such as robotics, lighting, and wireless sensor networks (WSNs) [12,13,14,15,16,17].

Among the various challenges in wastewater treatment, scenarios involving oil spills can be taken into account. Oil spills create a physical barrier that limits atmospheric gas exchange and reduces light penetration, particularly impacting the euphotic zone where photosynthesis occurs [18,19,20]. The reduction in oxygen availability is further exacerbated by the microbial oxidation processes that consume the remaining oxygen, leading to the formation of anoxic zones. These anoxic conditions hinder the activity of oxygen-dependent (oxygenotrophic) microorganisms, slowing down the degradation of hydrocarbons. Despite these challenges, successional microbial communities consisting of facultative aerobic or anaerobic hydrocarbon-degrading species can adapt to these conditions [21,22,23]. These bacteria form biofilms at the water–oil interface, which are three-dimensional structures that provide a protective and adaptive environment for microbial communities [24,25]. To further understand the complexity of these biofilms and the interactions within microbial communities, advanced techniques like water-in-oil droplet-based microfluidics offer a deeper understanding of microbial communities, allowing the detection of less dominant species that might be overshadowed by more prevalent hydrocarbon-degrading bacteria [26]. This approach could also help identify symbiotic interactions and key strains for improved energy conversion, enhancing the potential of microbial fuel cells in environmental remediation.

Biofilms play a crucial role in the degradation of hydrocarbons under anoxic conditions. The biofilm matrix facilitates nutrient availability and protects the bacteria from environmental stresses, enhancing their ability to degrade hydrocarbons effectively [27,28,29]. The existence of biofilms at the hydrocarbon–water interface has been documented, but the structural organization and the mechanisms enabling energy flow from the oil–water interface to the water column remain areas of active research.

In this study, we present an innovative MFC design that utilizes the electrogenic properties of hydrocarbonoclastic biofilms formed at the water–oil interface. By integrating these biofilms into the MFC architecture, we aim to take advantage of their role in electron transfer, exploiting them to achieve a self-sustaining cell that autonomously generates electrical energy. Moreover, MFCs based on hydrocarbonoclastic biofilms present a dual-functionality system that improves both sustainable power generation and advanced biosensing capabilities. These systems leverage the electrogenic properties of specialized biofilms to detect and to respond to environmental changes, making them highly effective for real-time monitoring and environmental remediation.

A unique feature of the MFC design we propose is that the biofilm naturally forms at the water–oil interface, which requires precise engineering to position the positive electrode there in order to optimize biofilm growth and electron transfer. Moreover, it functions autonomously without requiring external intervention or maintenance.

In this paper, we begin by introducing the background and objectives of the study, followed by a detailed explanation of the materials and methods, including the design of the microbial fuel cell and the approaches used for microbial community and electrochemical analyses. The results highlight key findings on biofilm formation, microbial community structure, and the electrochemical performance of the MFCs, with a particular focus on its long-term stability. Finally, we conclude by summarizing the insights gained from this research and discussing the potential applications of hydrocarbonoclastic biofilm-based MFCs.

## 2. Materials and Methods

### 2.1. MFC Design

For this study, a microbial fuel cell with a capacity of 100 L has been constructed using 0.8 cm thick plexiglass panels. The main structure consisted of four panels measuring 100 cm × 31 cm, reinforced at the corners with 1 cm × 1 cm plexiglass rods for additional stability. The assembly was secured using M4 hex head screws (RS Components, Milan, Italy) and brass inserts, along with “Anglosol 1200” (Anglo Adhesives & Services, Leicestershire, UK) acrylic adhesive. A 40 cm × 40 cm × 3 cm plexiglass sheet was used as the base, providing a stable foundation, with channels milled into it to facilitate drainage at the end of the experiments.

**Figure 1:**
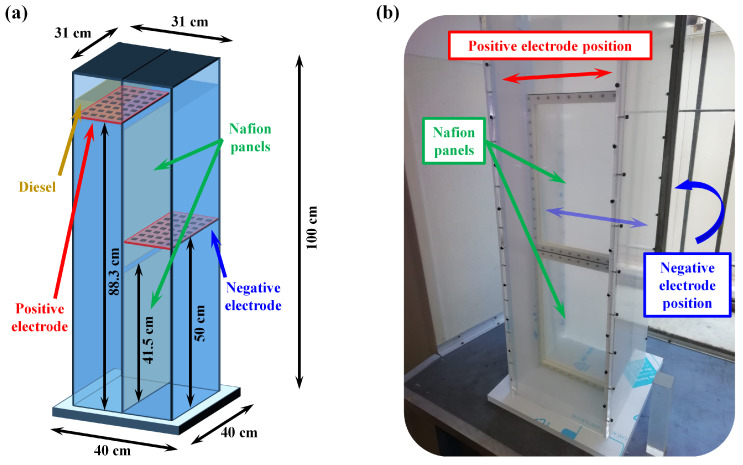
(**a**) Schematic representation of the 100-L MFC showing the placement of electrodes, Nafion panels, and diesel layer. (**b**) Photograph of the MFC during its construction, illustrating the assembly of the plexiglass panels and the Nafion panels. The positions where the positive and negative electrodes will subsequently be mounted are indicated.

Figure 1 shows a schematic representation of the 100-L MFC and a photograph of the MFC during its construction.

The scheme in Figure 1a provides a clear view of the internal configuration that ensures proper alignment and spacing of the components in order to optimize biofilm formation and electrochemical performance, while the photograph in Figure 1b shows one of the practical assembly steps.

Electrodes were mounted on 3D-printed Acrylonitrile butadiene styrene (ABS) frames resistant to hydrocarbons. These frames measured 11.8 cm × 29.2 cm × 0.5 cm, with an internal grid of 1 cm × 1 cm squares, providing an effective area of 10.8 cm × 28.2 cm. They were assembled using brass inserts and M4 screws, and four layers of carbon fabric were used for the electrodes. Electrical contact was ensured using 1 mm-diameter solid copper wire, which was shielded except at the contact points.

To form the two chambers, 3D-printed ABS frames were fitted with Nafion membranes (Ion Power Inc., Tyrone, PA, USA, Nafion^®^ Membrane N1100, 1100EW 10 mil-thick). These frames, measuring 29.6 cm × 41.5 cm × 0.5 cm with an active area of 23 cm × 35.5 cm, were sealed with O-rings and held in place by a plexiglass bar with adjustable clamps. An additional O-ring was placed between the frames to guarantee a tight seal. The two Nafion panels are clearly visible in the photograph in Figure 1b.

Based on our previous studies [30], the dimensions were chosen with the width being one-third of the height to promote biofilm formation. The positive electrode was positioned 88.3 cm from the bottom on the left side, while the negative electrode was placed 50 cm from the bottom on the right side.

In order to fill the stack, 100 L of the inoculum used in this study has been grown in lysogeny broth (LB) at room temperature (RT) and subsequently inoculated. Moreover, approximately 600 mL of diesel was added, forming a 2 cm layer above the positive electrode in order to create the right conditions for the biofilm formation. This thickness is sufficient to block oxygen diffusion from the atmosphere, ensuring the anoxic conditions necessary for the biofilm’s growth during the whole study.

Finally, two plexiglass covers, each measuring 31 cm × 13 cm, were employed to enclose the chambers.

### 2.2. Microbial Community Analysis

Understanding the composition and structure of the microbial community within the microbial fuel cell is crucial for evaluating its electrogenic performance. In this study, a detailed analysis of the microbial community was conducted through high-throughput 16S rRNA gene sequencing, bioinformatic processing, and electron microscopy of the biofilm, as detailed below.

#### 2.2.1. Sampling Site, Sample Processing, Total Microbiome DNA, and Gene Sequencing

The sampling of the inoculum used in this study was carried out at the tourist port of Fiumicino (Rome, Italy: 41°46′09.6″ N 12°13′32.5″ E). A 10-L sample was collected, stored in an ice box, and transported to the laboratory, where it was immediately processed following an ultrafiltration protocol as previously described [31]. The resulting retentate (50 mL) was aliquoted for laboratory investigations. After centrifugation, the pellet from 5 milliliters of the environmental sample was digested with lysozyme (Sigma-Aldrich, Burlington, MA, USA), and the same treatment was applied to the scraped pellet of the MFC samples. The total environmental DNA was then extracted using the Genomic DNA Tissue Kit (Machery-Nagel, Thermo Fisher Scientific, Waltham, MA, USA). The extracted environmental DNA was quantified both spectrophotometrically (NanoDrop, Thermo Fisher Scientific) and fluorometrically (Qubit, Thermo Fisher Scientific).

To analyze the microbial community, the V3-V4 hypervariable region of the prokaryotic ribosomal small subunit RNA (16S rRNA) gene was amplified from total environmental DNA using primers 341F (5′-TCGTCGGCAGCGTCAGATGTGTATAAGAGACAGCCTAC GGGNGGCWGCAG-3′) and 785R (5′-GTCTCGTGGGCTCGGAGATGTGTATAAGAGACA GGACTACHVGGGTATCTAATCC-3′), as previously reported by Klindworth et al. [32]. The 16S rRNA gene is highly conserved among all bacteria, making it an ideal target for studying microbial diversity. Its conserved regions allow for the alignment and comparison across different bacterial species, while its hypervariable regions provide the necessary specificity for identifying and differentiating between species. Amplicon sequencing of the 16S rRNA gene (550 bp) was then performed by the Bio-Fab Research Laboratory (Rome, Italy) using the MiSeq platform (MiSeqDx Reagent Kit v3 chemistry, Illumina, San Diego, CA, USA), with paired-end 2 × 300 bp reads.

#### 2.2.2. Bioinformatic Analysis of 16S rRNA Sequences

The quality of the 16S rRNA sequences generated in this study was evaluated using bioinformatics tools. The raw sequencing reads were processed by QIIME 2 (Quantitative Insights Into Microbial Ecology) v2023.5 using the *DADA2* (Divisive Amplicon Denoising Algorithm) to obtain high-quality ASV (Amplicon Sequence Variant) sequences. Representative ASV sequences were classified into taxa by a “home-made” naïve Bayesian Classifier based on the SILVA database v138.1 release. Taxonomic classifications were summarized in QIIME 2 and visualized using the R programming language with the ggplot2 package [33]. Metabolic reconstruction for the 16S rRNA datasets was conducted using PICRUSt v2.0.0 [34], which predicts metagenomes based on the Biological Observation Matrix (BIOM) abundance files and representative ASV sequences. These predicted metagenomes were further categorized into MetaCyc pathways using the MinPath algorithm [35], and their abundances were obtained.

Moreover, the evolutionary history of the predominant microbial genera identified in the MFC was inferred using the Maximum Likelihood method and the Kimura 2-parameter model [36]. A discrete Gamma distribution was applied to model evolutionary rate differences among sites, with 5 categories (+G, parameter = 0.5769), and some sites were allowed to remain evolutionarily invariable (+I, 23.70% sites). The analysis involved 283 nucleotide sequences. Evolutionary analyses were conducted in MEGA 11 [37].

#### 2.2.3. Electron Microscopy Analysis of Biofilm

Electron microscopy analysis was performed on the biofilm formed at the oil–water interface, i.e., on the graphite anode of the MFC. In particular, nine samples were collected after six months. These samples were allowed to adhere to poly-lysine treated glass coverslips for 3 h at room temperature and processed for scanning electron microscopy (SEM) analysis. The lipophilic substance produced by the bacterial cells under the same growth conditions was collected after centrifugation of the MFC planktonic biofilm to remove most cells from the floating lipophilic material. This sample was also allowed to adhere to poly-lysine treated glass coverslips for 3 h at RT and processed for SEM analysis. The samples were fixed in 2.5% glutaraldehyde in 0.1 M sodium cacodylate buffer overnight at 4 °C, post-fixed in 1% osmium tetroxide in the same buffer for 1 h at RT, and dehydrated through a graded ethanol series (30% to 100%). Ethanol was then gradually replaced with hexamethyldisilazane (HMDS) using a 1:1 ethanol:HMDS incubation for 30 minutes, followed by pure HMDS for 1 h. The samples were then allowed to dry under a chemical hood for 2 h, completely removing HMDS. Dried coverslips were mounted on stubs, gold-sputtered, and analyzed using a GeminiSEM450 field emission scanning electron microscope (Carl ZEISS, Oberkochen, Germany).

### 2.3. Electrochemical Measurements

The electrochemical measurements for the microbial fuel cell were performed using a customized multichannel measurement system designed for precise voltage and current monitoring, as previously described [38]. This system allows for accurate MFC performance characterization by employing high-precision analog-to-digital converters and transimpedance amplifiers.

Open-circuit voltage (OCV) and short-circuit current (SCC) measurements were carried out to assess the electrogenic properties of the biofilms formed within the MFC. For the analysis of power output and internal resistance, a series of digitally selectable resistors were applied to the MFC. Real-time voltage and current data were recorded, and the power output was calculated for each resistance. Subsequently, a least-squares regression method was used to fit the resulting data with a second-order curve, allowing the extraction of key performance parameters, including the maximum power point and internal resistance [38]. Moreover, long-term measurements were conducted to evaluate the total electrical power that the MFC can deliver.

**Figure 2:**
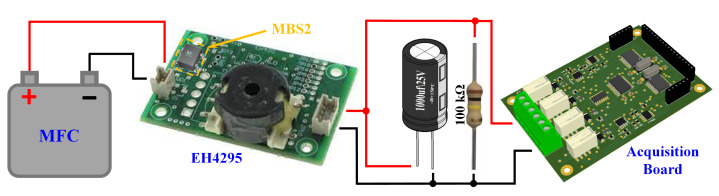
Experimental setup for long-term performance evaluations, including the MFC, EH4295 board, electrolytic capacitor, resistor, and acquisition board.

For long-term energy storage, we utilized the EH4295 commercial board from Advanced Linear Devices (ALD, Sunnyvale, CA, USA). This micropower step-up low-voltage booster module is ideal for use with MFCs due to its ability to operate efficiently with a minimum input voltage of 70 mV and a minimum input power of 2 μW. The EH4295 features a high input impedance of 950 Ω, which is suitable for MFCs with similar output resistances. The efficiency of the EH4295 ranges from 48% to 60%, optimized at around 0.75 V input voltage. The board provides both AC and DC outputs; for the latter, an MBS2 bridge rectifier from Vishay Intertechnology, Inc. (Malvern, PA, USA) was used, which can be mounted on a designated slot on the EH4295 board. To evaluate long-term performance, a 1 mF electrolytic capacitor was connected to the DC output of the board to act as a storage element. The total current delivered by the MFC over the long term was measured by discharging the output capacitor through a fixed 100 kΩ resistor mounted in parallel to the output. In this configuration, the output voltage across the capacitor was acquired using the same measurement system previously mentioned in voltage-measurement mode, as shown in Figure 2.

Data acquisition was managed using dedicated software, which provided real-time visualization and export of voltage and current values for further analysis. Furthermore, all measurements were conducted at a fixed temperature, maintaining the laboratory room at 24 °C.

## 3. Experiments

### 3.1. Biofilm and Microbial Community Characterization

To characterize the biofilm from a morphological and molecular perspective, the microbial community present on the anode was subjected to both scanning electron microscopy and molecular analyses.

#### 3.1.1. Biofilm Analysis

**Figure 3:**
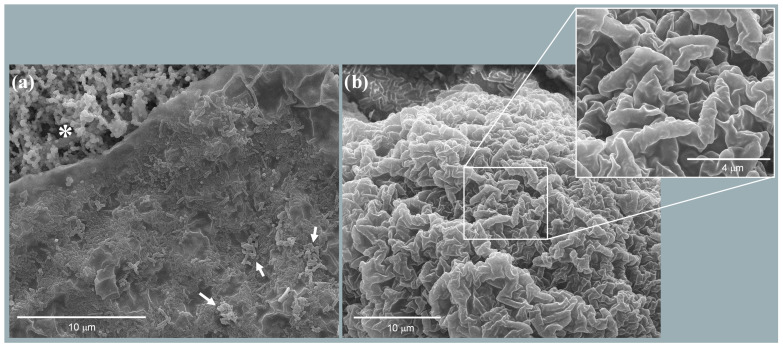
SEM micrographs of the membranous layer that develops at the oil–water interface of the MFC (3BF1; one out of nine samples): (**a**) The membranous layer above a lipid phase (*) where rod-shaped bacteria are clearly visible, embedded within the layer and aggregated on its surface (arrows), on the water side. (**b**) Lipid side of the layer with rod-shaped structures visible under the membranous layer (inset).

All collected biofilms were carefully extracted as previously described, scraped directly from the anode, and fixed for SEM observation. The SEM analysis focused on sample 3BF1, representative of the nine samples collected. As observed in previous studies on liquid MFCs [30], the electrogenic activation of the cell is accompanied by the formation of an aliphatic lipid barrier (Figure 3a), which separates the diesel-contaminated upper layer from the lower layer, enriched with hydrocarbonoclastic bacteria under the oily surface (Figure 3b). This amphipathic monolayer lipid barrier likely plays a crucial role in protecting the bacterial community from the harsh conditions created by the presence of oil while also facilitating the bioavailability of diesel molecules for microbial oxidation.

The water–diesel interface presents distinct physical properties, including viscosity, density, and gas permeability, which play a significant role in biofilm formation and stability. Diesel, with its relatively high viscosity and lower gas permeability compared to other oil types, such as fluorinated oils like HFE7500, creates a different environment for biofilm development. Indeed, fluorinated oils, known for their higher gas permeability, could facilitate greater oxygen transfer at the interface, potentially impacting microbial activity and electrogenic performance. Future studies will explore how these variations in oil–water interfaces can influence the overall efficiency of the MFC system.

#### 3.1.2. Microbial Community Characterization

**Figure 4:**
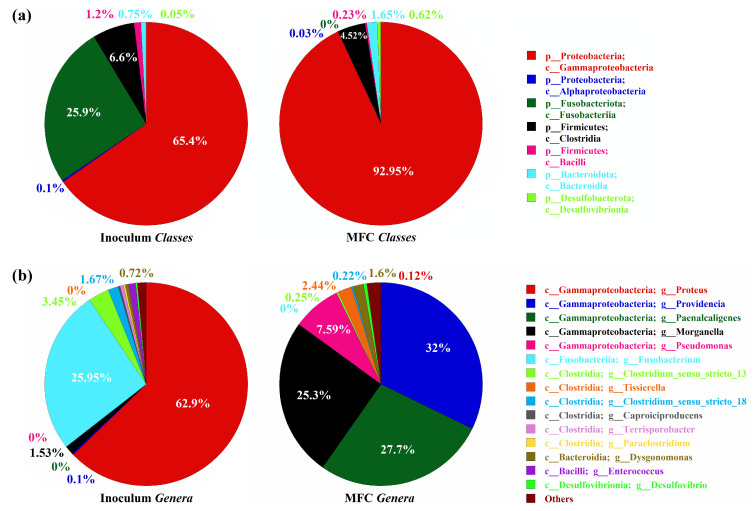
Diagram showing the percentage of microbial Classes (**a**) and Genera (**b**) found in the initial inoculum and in the MFC at the end of the experiment. For the MFC data, the values reported in the pie charts were obtained by averaging the analyses performed on the 9 samples collected.

The microbial inoculum administered in the MFC was collected from a small tourist port frequented by boats. The inoculum was predominantly composed of Proteobacteria (65.5%), Fusobacteriota (26%), Firmicutes (7.7%), and a mixed set of minor components, including approximately 1% Bacteroidota. The community was genetically characterized by sequencing the 16S rRNA gene, which revealed a significant increase in the proportion of *Gammaproteobacteria* in the anode microbiome as the experiment progressed. This subclass, known for its extensive hydrocarbonoclastic capabilities, increased from 65.4% in the original inoculum to over 90% in the MFC, as shown in Figure 4a.

Figure 4 illustrates the composition of microbial Classes and Genera found in the inoculated sample and in the MFC at the end of the experiment. This observation aligns with previous studies indicating the crucial role of *Gammaproteobacteria* in hydrocarbon degradation, particularly during events like the Deepwater Horizon oil spill [39], where this class was found to be notably enriched due to its ability to degrade various hydrocarbons, including alkanes and polycyclic aromatic hydrocarbons (PAHs) [40,41].

**Figure 5:**
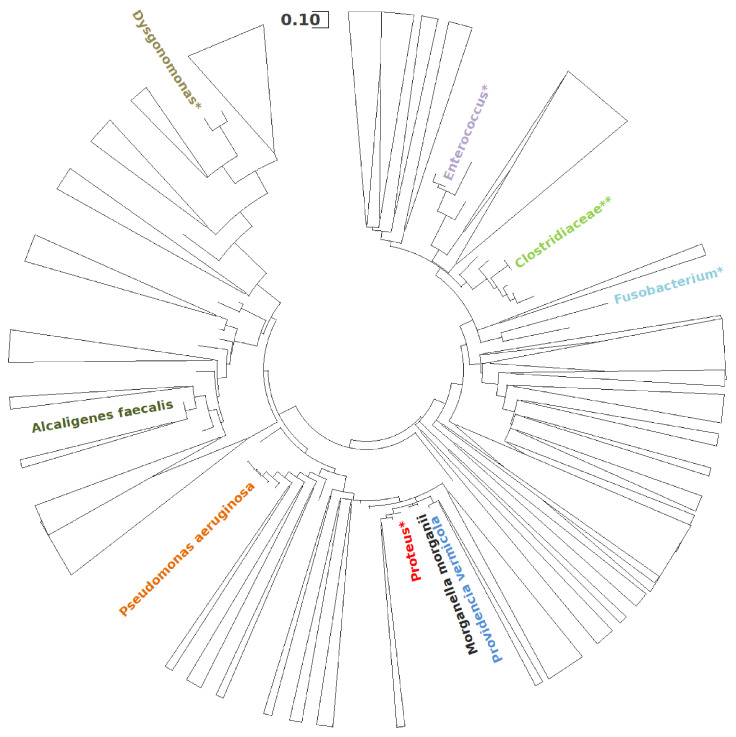
Phylogenetic tree illustrating the evolutionary relationships among the predominant microbial families, genera, and species identified in the MFC. The tree structure combines family-level (**), genus-level (*), and species-level insights, providing a comprehensive overview of the microbial community composition.

This analysis of the microbial composition suggests a strong selection of specific genera capable of thriving in the oil-contaminated environment of the MFC. To better understand the evolutionary relationships between these predominant taxa, a comprehensive phylogenetic analysis was conducted, illustrating the evolutionary affinities among the members of the microbial community. The resulting phylogenetic tree, shown in Figure 5, illustrates the evolutionary relationships across 283 nucleotide sequences. The taxa included in this analysis represent key members of the microbial community, such as *Alcaligenes faecalis*, *Pseudomonas aeruginosa*, *Providencia vermicola*, and *Morganella morganii*, which could contribute to the electrogenic and hydrocarbonclastic activity of the system.

As the experiment progressed, a clear adaptive convergence was observed in the anodic community, highlighting the selective enrichment of specific genera capable of thriving in the oil-contaminated environment of the MFC. Notably, genera such as *Proteus*, which were present in the original inoculum, were almost completely replaced by genera better suited to the conditions within the MFC, such as *Providencia*, *Paenalcaligenes*, *Morganella*, and *Pseudomonas*. This shift underscores the dynamic nature of the microbial community as it adapted to the MFC environment.

For instance, *Alcaligenes faecalis*, a member of the *Proteobacteria* with specialized hydrocarbon-degrading abilities [42], was notably enriched (99.3% of the genus *Paenalcaligenes*, corresponding to 27.51% of the total genome). This bacterium, often found in oil-contaminated ecosystems, plays a key role in breaking down complex hydrocarbons, complementing the activities of other specialized microorganisms such as *Pseudomonas aeruginosa* [43]. Furthermore, *Providencia vermicola* became another of the dominant species, reaching a mean abundance of 31.9% and a peak abundance of approximately 41% in the SEM-analyzed sample. Alongside *P. aeruginosa*, *P. vermicola* demonstrated the ability to utilize various hydrocarbons, including diesel, gasoline, kerosene, and motor oil, for growth [44]. Additionally, *Morganella morganii*, known for its ability to degrade PAHs, was consistently identified across all samples with an average prevalence of 17.5%. The presence of *M. morganii*, which has demonstrated high degradative activity toward naphthalene, highlights its role in the comprehensive degradation of both long- and short-chain alkanes within the MFC [45].

The genus *Dysgonomonas*, although present in smaller proportions with an average representation of about 1.6%, was another notable component of the community. Recognized for its crude-oil degrading potential, *Dysgonomonas* contributed to the microbial consortia’s overall functionality, as demonstrated in previous studies focused on oil and wastewater treatment [46].

Overall, the diversity and specialization of the hydrocarbon-degrading genera observed in the MFC indicate a highly optimized microbial community capable of effectively metabolizing the available hydrocarbons. This specialization facilitated the breakdown of complex organic pollutants and significantly contributed to the stability and efficiency of the MFC over time, as these genera played a central role in sustaining the electrogenic activity of the system.

In addition to energy generation, the MFC demonstrated a measurable reduction in the diesel layer over the course of six months. Starting from an initial volume of 600 mL of diesel, approximately 420 mL was degraded by the biofilm, as evidenced by the cumulative consumption observed during the experiment. This reduction suggests that the biofilm can degrade hydrocarbons, confirming its potential for applications in wastewater remediation. While further studies are necessary to optimize and accelerate this process, these initial results indicate that the system holds promise for treating environments contaminated with oil-based pollutants.

### 3.2. Electrochemical Performance and Electrical Power Generation

The electrochemical performance of the microbial fuel cell was evaluated through a series of measurements, including current, voltage, and power output, after waiting six months for the biofilm to form and fully cover the area of the positive electrode. These aimed to determine the efficiency and capacity of the MFC in generating electrical energy from organic substrates.

The peak power that the cell is able to deliver was assessed by performing real-time measurements using a protocol of alternating charge and discharge phases. A set of 12 resistive loads was employed, as shown in the figures below. Each charge phase lasted 3 min, sufficient to reach the pseudo-steady-state of the cell following the discharge phase, while each discharge phase lasted 0.5 s.

**Figure 6:**
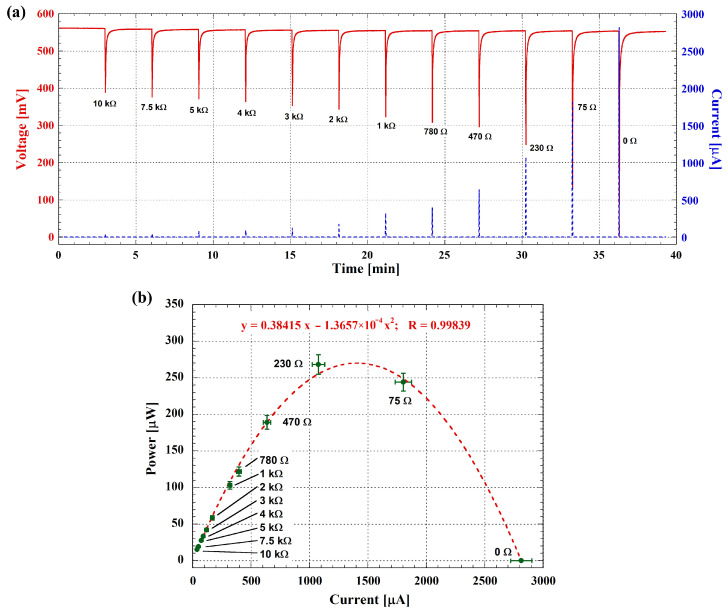
(**a**) Voltage (left y-axis) and current (right y-axis) measurements of the MFC. The resistances used for each discharging step are reported in the figure. The data are obtained by averaging 5 measurements performed at 1-h intervals on the same day. (**b**) Peak power output as a function of current. Experimental data are shown as symbols, while the fitting curve is represented by the dashed line. Error bars, both for current and power values, indicate 5 different measurements performed at 1-h intervals on the same day.

Figure 6a shows the voltage and current of the cell throughout the experiment. The OCV, which is about 560 mV, can be observed as the pseudo-steady-state voltage after each discharge phase. The left y-axis illustrates the cell voltage, while the right one displays the corresponding current peaks for various resistive loads, with the final peak representing the SCC, whose value is about 2800 μA.

The peak power of the cell as a function of the current was inferred from the acquired data represented in Figure 6a and reported in Figure 6b, where the experimental data are shown as symbols and the fitting curve is represented by the dashed line. The maximum peak power output of the cell is 270 μW, corresponding to a dynamic output resistance of 136.6 Ω as derived from the fitting equation.

**Figure 7:**
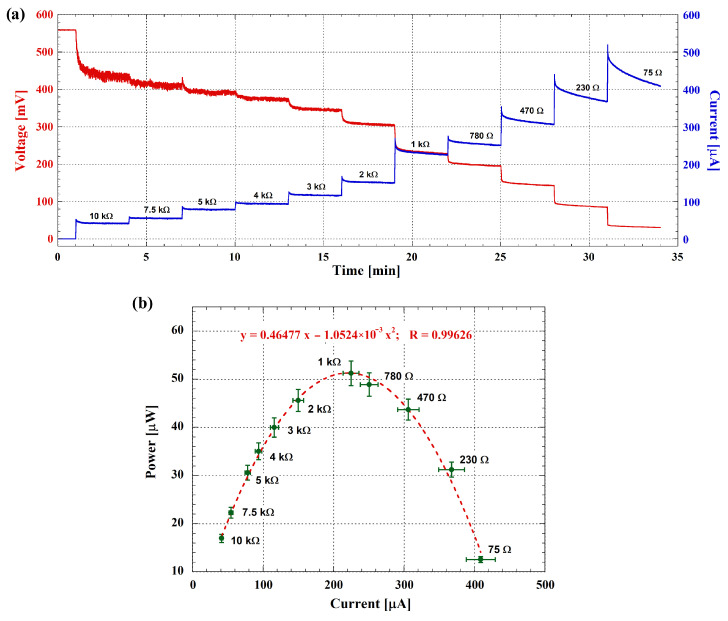
(**a**) Voltage (left axis) and current (right axis) evolution of the MFC during the static output power characterization. (**b**) Static power output as a function of current. Experimental data are shown as symbols, with the fitting curve represented by the dashed line. Error bars, both for current and power values, indicate 5 different measurements performed at 1-h intervals on the same day.

Static power measurements followed a different protocol. In this case, the cell was discharged across the same resistive loads as before (except for the 0 Ω load), but each discharge lasted 3 min to reach a quasi-steady-state current, and there was no charging phase between discharges. Figure 7a shows the voltage and current of the cell during these static power measurements. The red curve illustrates the cell voltage, while the blue one displays the corresponding current evolution for the chosen resistive loads.

As observed in Figure 7a, the red curve (i.e., the voltage across the cell) exhibits a high noise level during the initial minutes of acquisition, which decreases with lower resistive load values. This behavior can be explained by considering the operation of the measuring system already described [38]. In particular, during the discharge phase, the system measures the current flowing out of the MFC, while the voltage across it is inferred since the resistive load value is known. Therefore, given a constant level of noise associated with the measured current, the noise in the voltage is higher when the resistance connected to the cell is higher.

The static output power of the cell was then determined from the data of Figure 7a and shown as a function of the current. Figure 7b presents the static power data, with the experimental data represented by symbols and the fitting curve by the dashed line. The internal resistance of the cell, extracted from these measurements, is about 1.05 kΩ, as seen from the fitting equation. Moreover, the maximum power point for the presented MFC is about 51.5 μW.

The power output of our MFC, lower than typically reported in hydrocarbon-degrading MFCs, is consistent with the system’s unique design. As this system operates under fully anoxic conditions and relies on a naturally forming biofilm without maintenance, it produces power one to two orders of magnitude lower than comparable MFCs [47,48,49,50]. Nevertheless, in the next section, we demonstrate that this power output is sufficient to support low-power applications such as remote sensing and environmental monitoring, which was the primary objective of this study.

### 3.3. Long-Term Stability and Possible Applications

The mechanical stability of the biofilm at the water–oil interface is an essential factor in the long-term operation of this MFC system. The biofilm forms directly on the positive electrode, which is positioned precisely at this interface, where the interfacial tension between the oil and water phases provides mechanical support. Since the system operates under static conditions, without fluid flow, and with a constant temperature, the biofilm remains stable throughout the experiment. These controlled conditions prevent disturbances to the biofilm, ensuring its structural integrity and consistent electrogenic performance over the six-month experimental period without the need for external intervention.

The long-term stability of the microbial fuel cell was assessed by conducting continuous voltage measurements over an extended period. As reported above, the measurements were performed using the EH4295 commercial board connected to a 1 mF electrolytic capacitor for energy storage. The capacitor was subsequently discharged using a fixed value resistor, and the voltage across it was acquired using the previously described measurement system to determine the total current delivered by the MFC over the long term.

**Figure 8:**
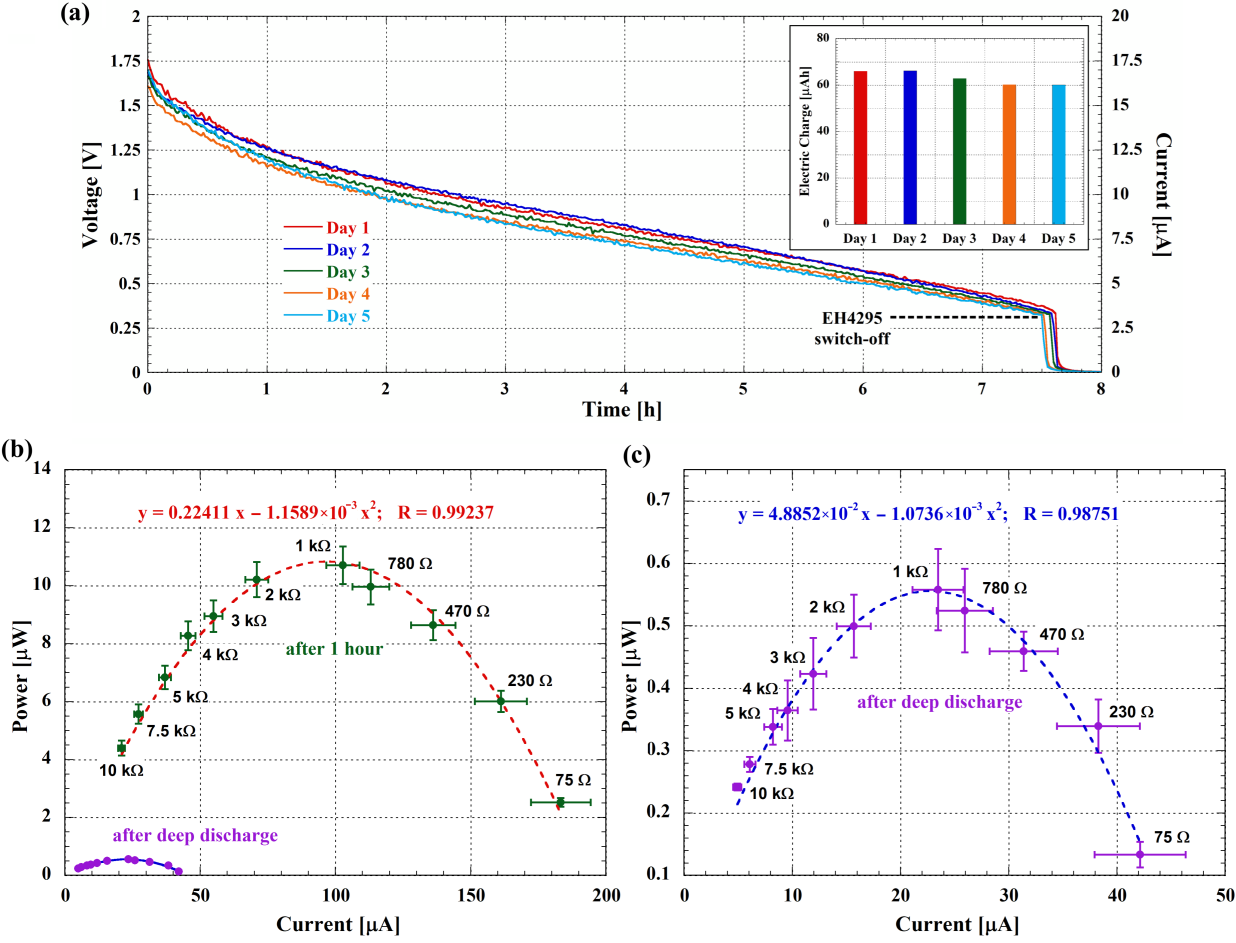
(**a**) Voltage (left y-axis) and corresponding current (right y-axis) across the 100 kΩ resistor over a 5-day period, showing 8-h discharges between 16-h recovery cycles. The EH4295 board switches off after approximately 7.5 h, indicating the deep discharge status. The inset shows the integrated electric charge value for each day. (**b**) Power output of the MFC immediately after the 8-h measurement cycles (purple symbols with blue fitting curve) and one hour later (green symbols with red fitting curve and corresponding equation). (**c**) Zoomed view of the power output immediately after the 8-h measurement cycles and related fitting equation. Error bars, both for current and power values and for both curves, indicate the 5 different acquisitions performed in the considered days of measurements.

The cell was able to deliver power for 7.5 h before going through the deep discharge. This occurs when the EH4295 board switches off as the electrical power input drops below its operational threshold. Measurements were taken daily at the same time, with each acquisition lasting 8 h. After a recovery period of 16 h, the cell was discharged again, repeating this cycle for 5 days. Figure 8a shows the voltage across the 100 kΩ resistor during these cycles. The data indicate stable performance with consistent recovery and discharge cycles over the 5-day period, and the slight variations in voltage over different days suggest minor fluctuations in performance, which are typical for bio-electrochemical systems.

The left y-axis in Figure 8a shows the voltage across the resistor-capacitor parallel, while the right y-axis indicates the corresponding current. This dual-axis representation provides a clear view of both parameters, highlighting their relationship during the discharge cycles. The histogram placed as an inset in Figure 8a shows the stored electric charge, calculated by integrating the current over the discharge time for each day, and underscores that the average value of the storage charge is about 63 μAh. Furthermore, starting from the data reported in Figure 8a, it is possible to obtain the delivered power for the 8-h acquisition period, and by integrating this power over the discharge time, the total stored energy can be evaluated for each day. The average energy stored per day is about 60 μWh, corresponding to 216 mJ.

Additionally, we measured the power output of the cell immediately after deep discharge and again one hour later. Figure 8b,c present these measurements, highlighting the recovery capability of the MFC. The data demonstrate that the cell can restore a significant portion of its power output after a short recovery period, further indicating its long-term stability.

The measured average output energy of approximately 60 μWh per day indicates that the MFC can support low-power applications over extended periods. Indeed, considering, for instance, the study by Yamashita et al. [51], both short- and long-range data transmission can be achieved. Yamashita et al. conducted various environmental measurements, including CO_2_ levels, temperature, and humidity, to demonstrate the viability of MFCs for powering environmental sensors and wireless data transmission systems. They explored the use of LoRa technology for long-range communication and Bluetooth Low Energy (BLE) for short-range data transmission.

For the latter, they demonstrated that an average current of 170 μA flowing for 6 ms and an energy of 3.06 μJ were required for a single combined measurement of both temperature and humidity and BLE transmission. This low energy requirement suggests that our MFC could support a significantly higher number (thousands) of short-range data transmissions daily, showcasing its versatility and potential for various remote sensing and monitoring applications.

For long-range data transmission, the cited study showed that their MFC could support wireless data transmission using LoRa technology. The LoRa modules employed require an average current of 90 mA for 250 ms, resulting in an energy consumption of 68 mJ per transmission. Given that our can store approximately 216 mJ per day, it is feasible to perform multiple long-range data transmissions daily, making it suitable also for long-range remote environmental monitoring applications.

The obtained results highlight our MFC’s efficiency in energy utilization and underscore its suitability for both short-range and long-range remote environmental monitoring applications. This dual capability demonstrates a clear advantage over existing solutions, offering a more robust and adaptable platform for a wide range of remote sensing scenarios.

## 4. Conclusions

In this study, we presented a novel microbial fuel cell design with a capacity of 100 L that leverages hydrocarbonoclastic biofilms formed at the water–oil interface to enhance both renewable energy generation and wastewater remediation. The microbial community analysis revealed a substantial enrichment of *Gammaproteobacteria*, which increased from 65.4% in the initial inoculum to over 90% in the biofilms formed on the anode.

The MFC developed in this study achieved a peak power output of 270 μW, with a corresponding internal resistance of 136.6 Ω, and demonstrated stable operation over extended periods. Static power measurements revealed a maximum output of 51.5 μW with an internal resistance of 1.05 kΩ. Additionally, the system’s ability to recover and maintain performance after deep discharge was confirmed, with the MFC capable of delivering power for up to 7.5 h before requiring a recovery period. Over a five-day testing period, the MFC consistently stored an average of 216 mJ per day, sufficient to support low-power applications such as remote sensing and environmental monitoring.

These results suggest that hydrocarbonoclastic biofilm-based MFCs offer a promising dual-functionality system that contributes to sustainable energy generation and addresses environmental challenges related to wastewater treatment and oil spill remediation. The ability to generate power while simultaneously degrading hydrocarbons highlights the potential of this technology for use in autonomous sensor networks, where reliable and sustainable power sources are essential. This MFC design is distinct in its ability to operate autonomously, utilizing a biofilm that forms naturally at the water–oil interface. This feature eliminates the need for active maintenance or intervention, making it suitable for applications requiring long-term stability and minimal external input. Although the power output is lower than other hydrocarbon-degrading MFCs, the system’s ability to support remote sensing applications demonstrates its potential for environmental monitoring and remediation. Furthermore, the electrogenic capabilities of hydrocarbonoclastic biofilms suggest that this MFC design can be effectively integrated as a biosensor for detecting environmental pollutants, such as hydrocarbons, in real time. This dual functionality underscores the system’s applicability in both energy production and environmental monitoring.

Future research should focus on scaling up this technology and exploring its integration into various environmental applications. This study provides a foundation for developing advanced MFCs that can serve not only as efficient energy generators but also as effective tools for environmental remediation. By combining these dual capabilities, our discovery paves the way for groundbreaking solutions that address both renewable energy needs and environmental sustainability challenges. Furthermore, exploring potential symbiotic interactions during biofilm formation, such as by designing experiments with an artificial community of two different species or by loading *Gammaproteobacteria* alone, could yield further insights. This may help determine whether similar or higher levels of energy generation can be achieved with a simplified community structure. In summary, this innovative approach positions MFCs as a promising and versatile technology for future applications in energy production, waste management, environmental biosensing, and ecological preservation, offering a valuable contribution to a more sustainable and resilient world while leaving room for further exploration of alternative solutions.

## Data Availability

The original contributions presented in the study are included in the article. Further inquiries can be directed to the corresponding authors.

## Abbreviations

The following abbreviations are used in this manuscript:

ABS	Acrylonitrile Butadiene Styrene
ASV	Amplicon Sequence Variant
BIOM	Biological Observation Matrix
BLE	Bluetooth Low Energy
DADA	Divisive Amplicon Denoising Algorithm
DNA	Deoxyribonucleic Acid
HMDS	Hexamethyldisilazane
LB	Lysogeny Broth
LoRa	Long Range
MFC	Microbial Fuel Cell
OCV	Open-Circuit Voltage
PAH	Polycyclic Aromatic Hydrocarbon
PCR	Polymerase Chain Reaction
QIIME	Quantitative Insights Into Microbial Ecology
RNA	Ribonucleic Acid
RT	Room Temperature
SCC	Short-Circuit Current
SEM	Scanning Electron Microscopy

## References

[1] Wang J., Ren K., Zhu Y., Huang J., Liu S. (2022). A review of recent advances in microbial fuel cells: Preparation, operation, and application. BioTech.

[2] Boas J.V., Oliveira V.B., Simões M., Pinto A.M. (2022). Review on microbial fuel cells applications, developments and costs. J. Environ. Manag..

[3] Roy H., Rahman T.U., Tasnim N., Arju J., Rafid M.M., Islam M.R., Pervez M.N., Cai Y., Naddeo V., Islam M.S. (2023). Microbial fuel cell construction features and application for sustainable wastewater treatment. Membranes.

[4] Jatoi A.S., Hashmi Z., Mazari S.A., Mubarak N.M., Karri R.R., Ramesh S., Rezakazemi M. (2022). A comprehensive review of microbial desalination cells for present and future challenges. Desalination.

[5] Kurniawan T.A., Othman M.H.D., Liang X., Ayub M., Goh H.H., Kusworo T.D., Mohyuddin A., Chew K.W. (2022). Microbial fuel cells (MFC): A potential game-changer in renewable energy development. Sustainability.

[6] Kižys K., Zinovičius A., Jakštys B., Bružaitė I., Balčiūnas E., Petrulevičienė M., Ramanavičius A., Morkvėnaitė-Vilkončienė I. (2023). Microbial biofuel cells: Fundamental principles, development and recent obstacles. Biosensors.

[7] Prathiba S., Kumar P.S., Vo D.V.N. (2022). Recent advancements in microbial fuel cells: A review on its electron transfer mechanisms, microbial community, types of substrates and design for bio-electrochemical treatment. Chemosphere.

[8] Andriukonis E., Celiesiute-Germaniene R., Ramanavicius S., Viter R., Ramanavicius A. (2021). From microorganism-based amperometric biosensors towards microbial fuel cells. Sensors.

[9] Bhowmik D., Chetri S., Enerijiofi K.E., Naha A., Kanungo T.D., Shah M.P., Nath S. (2023). Multitudinous approaches, challenges and opportunities of bioelectrochemical systems in conversion of waste to energy from wastewater treatment plants. Clean. Circ. Bioecon..

[10] Patel A.K., Singhania R.R., Albarico F.P.J.B., Pandey A., Chen C.W., Dong C.D. (2022). Organic wastes bioremediation and its changing prospects. Sci. Total Environ..

[11] Ochieng R., Gebremedhin A., Sarker S. (2022). Integration of waste to bioenergy conversion systems: A critical review. Energies.

[12] Borja-Maldonado F., López Zavala M.Á. (2023). Assessment of Graphite, Graphene, and Hydrophilic-Treated Graphene Electrodes to Improve Power Generation and Wastewater Treatment in Microbial Fuel Cells. Bioengineering.

[13] Garbini G.L., Barra Caracciolo A., Grenni P. (2023). Electroactive bacteria in natural ecosystems and their applications in microbial fuel cells for bioremediation: A review. Microorganisms.

[14] Mahmoud R.H., Gomaa O.M., Hassan R.Y. (2022). Bio-electrochemical frameworks governing microbial fuel cell performance: Technical bottlenecks and proposed solutions. RSC Adv..

[15] Verma M., Mishra V. (2021). Recent trends in upgrading the performance of yeast as electrode biocatalyst in microbial fuel cells. Chemosphere.

[16] Gajda I., Greenman J., Ieropoulos I. (2020). Microbial Fuel Cell stack performance enhancement through carbon veil anode modification with activated carbon powder. Appl. Energy.

[17] Srivastava R.K., Boddula R., Pothu R. (2022). Microbial fuel cells: Technologically advanced devices and approach for sustainable/renewable energy development. Energy Convers. Manag. X.

[18] Bacosa H.P., Ancla S.M.B., Arcadio C.G.L.A., Dalogdog J.R.A., Ellos D.M.C., Hayag H.D.A., Jarabe J.G.P., Karim A.J.T., Navarro C.K.P., Palma M.P.I. (2022). From surface water to the deep sea: A review on factors affecting the biodegradation of spilled oil in marine environment. J. Mar. Sci. Eng..

[19] Silva I.A., Almeida F.C., Souza T.C., Bezerra K.G., Durval I.J., Converti A., Sarubbo L.A. (2022). Oil spills: Impacts and perspectives of treatment technologies with focus on the use of green surfactants. Environ. Monit. Assess..

[20] Krek E.V., Krek A.V., Kostianoy A.G. (2021). Chronic oil pollution from vessels and its role in background pollution in the southeastern baltic sea. Remote Sens..

[21] Zhou Y., Wang Y., Yao S., Zhao X., Kong Q., Cui L., Zhang H. (2024). Driving mechanisms for the adaptation and degradation of petroleum hydrocarbons by native microbiota from seas prone to oil spills. J. Hazard. Mater..

[22] Yan L., Hui N., Simpanen S., Tudeer L., Romantschuk M. (2020). Simulation of microbial response to accidental diesel spills in basins containing brackish sea water and sediment. Front. Microbiol..

[23] Truskewycz A., Gundry T.D., Khudur L.S., Kolobaric A., Taha M., Aburto-Medina A., Ball A.S., Shahsavari E. (2019). Petroleum hydrocarbon contamination in terrestrial ecosystems—fate and microbial responses. Molecules.

[24] Ehiosun K.I., Godin S., Urios L., Lobinski R., Grimaud R. (2022). Degradation of long-chain alkanes through biofilm formation by bacteria isolated from oil-polluted soil. Int. Biodeterior. Biodegrad..

[25] Khalid F.E., Lim Z.S., Sabri S., Gomez-Fuentes C., Zulkharnain A., Ahmad S.A. (2021). Bioremediation of diesel contaminated marine water by bacteria: A review and bibliometric analysis. J. Mar. Sci. Eng..

[26] Terekhov S.S., Smirnov I.V., Malakhova M.V., Samoilov A.E., Manolov A.I., Nazarov A.S., Danilov D.V., Dubiley S.A., Osterman I.A., Rubtsova M.P. (2018). Ultrahigh-throughput functional profiling of microbiota communities. Proc. Natl. Acad. Sci. USA.

[27] Mishra S., Huang Y., Li J., Wu X., Zhou Z., Lei Q., Bhatt P., Chen S. (2022). Biofilm-mediated bioremediation is a powerful tool for the removal of environmental pollutants. Chemosphere.

[28] Mahto K.U., Kumari S., Das S. (2022). Unraveling the complex regulatory networks in biofilm formation in bacteria and relevance of biofilms in environmental remediation. Crit. Rev. Biochem. Mol. Biol..

[29] Sahreen S., Mukhtar H., Imre K., Morar A., Herman V., Sharif S. (2022). Exploring the function of quorum sensing regulated biofilms in biological wastewater treatment: A review. Int. J. Mol. Sci..

[30] D’Ugo E., Bertuccini L., Spadaro F., Giuseppetti R., Iosi F., Santavenere F., Giuliani F., Gricia M., Rodomonte A., Lovecchio N. (2021). Electrogenic and hydrocarbonoclastic biofilm at the oil-water interface as microbial responses to oil spill. Water Res..

[31] D’Ugo E., Marcheggiani S., Fioramonti I., Giuseppetti R., Spurio R., Helmi K., Guillebault D., Medlin L.K., Simeonovski I., Boots B. (2016). Detection of human enteric viruses in freshwater from European countries. Food Environ. Virol..

[32] Klindworth A., Pruesse E., Schweer T., Peplies J., Quast C., Horn M., Glöckner F.O. (2013). Evaluation of general 16S ribosomal RNA gene PCR primers for classical and next-generation sequencing-based diversity studies. Nucleic Acids Res..

[33] Wickham H. (2009). ggplot2: Elegant Graphics for Data Analysis.

[34] Douglas G.M., Maffei V.J., Zaneveld J., Yurgel S.N., Brown J.R., Taylor C.M., Huttenhower C., Langille M.G. (2019). PICRUSt2: An improved and extensible approach for metagenome inference. BioRxiv.

[35] Langille M.G., Zaneveld J., Caporaso J.G., McDonald D., Knights D., Reyes J.A., Clemente J.C., Burkepile D.E., Vega Thurber R.L., Knight R. (2013). Predictive functional profiling of microbial communities using 16S rRNA marker gene sequences. Nat. Biotechnol..

[36] Kimura M. (1980). A simple method for estimating evolutionary rates of base substitutions through comparative studies of nucleotide sequences. J. Mol. Evol..

[37] Tamura K., Stecher G., Kumar S. (2021). MEGA11: Molecular evolutionary genetics analysis version 11. Mol. Biol. Evol..

[38] Lovecchio N., Di Meo V., Pietrelli A. (2023). Customized Multichannel Measurement System for Microbial Fuel Cell Characterization. Bioengineering.

[39] Beyer J., Trannum H.C., Bakke T., Hodson P.V., Collier T.K. (2016). Environmental effects of the Deepwater Horizon oil spill: A review. Mar. Pollut. Bull..

[40] Gutierrez T. (2019). Marine, aerobic hydrocarbon-degrading gammaproteobacteria: Overview. Taxonomy, Genomics and Ecophysiology of Hydrocarbon-Degrading Microbes.

[41] Zhou Z., Liu Y., Pan J., Cron B.R., Toner B.M., Anantharaman K., Breier J.A., Dick G.J., Li M. (2020). Gammaproteobacteria mediating utilization of methyl-, sulfur-and petroleum organic compounds in deep ocean hydrothermal plumes. ISME J..

[42] Zeng C., Li Y., Lu A., Ding H., Wang X., Wang C. (2012). Electrochemical interaction of a heterotrophic bacteria Alcaligenes faecalis with a graphite cathode. Geomicrobiol. J..

[43] Yang H.H., Jun H.K., Jung Y.J., Choi B.K. (2014). Enterococcus faecalis activates caspase-1 leading to increased interleukin-1 beta secretion in macrophages. J. Endod..

[44] Kumar R., De M. (2023). Enhanced degradation of petroleum hydrocarbons by Klebsiella michiganensis RK and Acinetobacter baumannii IITG19 isolated from local soil sources. Int. J. Environ. Sci. Technol..

[45] Atlas R.M. (1981). Microbial degradation of petroleum hydrocarbons: An environmental perspective. Microbiol. Rev..

[46] Huang Z., He X., Nye C., Bagley D., Urynowicz M., Fan M. (2021). Effective anaerobic treatment of produced water from petroleum production using an anaerobic digestion inoculum from a brewery wastewater treatment facility. J. Hazard. Mater..

[47] Oveisi F., Fallah N., Nasernejad B. (2021). Biodegradation of synthetic wastewater containing styrene in microbial fuel cell: Effect of adaptation of microbial community. Fuel.

[48] Umar M.F., Rafatullah M., Abbas S.Z., Ibrahim M.N.M., Ismail N. (2021). Bioelectricity production and xylene biodegradation through double chamber benthic microbial fuel cells fed with sugarcane waste as a substrate. J. Hazard. Mater..

[49] Nandy A., Radović J.R., Novotnik B., Sharma M., Larter S.R., Thangadurai V. (2020). Investigation of crude oil degradation using metal oxide anode-based microbial fuel cell. Bioresour. Technol. Rep..

[50] Li X., Zheng R., Zhang X., Liu Z., Zhu R., Zhang X., Gao D. (2019). A novel exoelectrogen from microbial fuel cell: Bioremediation of marine petroleum hydrocarbon pollutants. J. Environ. Manag..

[51] Yamashita T., Hayashi T., Iwasaki H., Awatsu M., Yokoyama H. (2019). Ultra-low-power energy harvester for microbial fuel cells and its application to environmental sensing and long-range wireless data transmission. J. Power Sources.

